# Transcatheter Arterial Chemoembolization in Combination With High-Intensity Focused Ultrasound for Intermediate and Advanced Hepatocellular Carcinoma: A Meta-Analysis

**DOI:** 10.3389/fonc.2022.797349

**Published:** 2022-03-28

**Authors:** Yun-Bing Wang, Rong Ma, Zhi-Biao Wang, Qiu-Ling Shi, Lian Zhang, Wen-Zhi Chen, Jian-Ping Gong, Jin Bai

**Affiliations:** ^1^ State Key Laboratory of Ultrasound in Medicine and Engineering, College of Biomedical Engineering, Chongqing Medical University, Chongqing, China; ^2^ Department of Hepatobiliary Surgery, The Second Affiliated Hospital of Chongqing Medical University, Chongqing, China; ^3^ School of Public Health and Management, Chongqing Medical University, Chongqing, China

**Keywords:** transcatheter arterial chemoembolization, high-intensity focused ultrasound, combination, hepatocellular carcinoma, meta-analysis

## Abstract

**Background:**

The study was conducted to explore whether high-intensity focused ultrasound (HIFU) can improve the effect of transcatheter arterial chemoembolization (TACE) in intermediate and advanced hepatocellular carcinoma (HCC).

**Methods:**

PubMed, Embase, Cochrane Library, Web of Science, Wanfang Data, CQVIP, China National Knowledge Infrastructure (CNKI), and Chinese Biomedical (CBM) databases were searched for randomized controlled trials (RCTs) comparing the effect of TACE in combination with HIFU group (group A) to TACE alone group (group B) in treating intermediate and advanced HCC. The primary outcomes were overall survival (OS) rate and tumor response rate. The odds ratio (OR) and 95% confidence interval (CI) for each study were calculated and then pooled with fixed effects model or random effects model. Sensitivity analyses and subgroup analyses were conducted. A publication bias was also evaluated.

**Results:**

After literature selection, eleven RCTs involving 803 patients were included in this meta-analysis. This meta-analysis revealed that group A was associated with an increased 6-month OS rate (OR = 0.20), 12-month OS rate (OR = 0.23), 24-month OS rate (OR = 0.32), and overall response rate (WHO criterion, OR = 0.22; RECIST criterion, OR = 0.30). Furthermore, subgroup analyses showed no bias in the result. Given the limited number of studies that reported major complications, no additional meta-analysis of complication was conducted. Despite no special treatment, any complication following HIFU treatment was found to subside within 3-7 days.

**Conclusion:**

TACE in combination with HIFU is associated with increased OS and tumor response in intermediate and advanced HCC. Current evidence supports the use of HIFU after TACE treatment in intermediate and advanced HCC.

## Introduction

Primary liver cancer is the sixth most commonly diagnosed cancer and the third leading cause of cancer death worldwide in 2020 (1). Hepatocellular carcinoma (HCC) accounts for 75-85% of all liver cancer cases. As the majority of HCC patients are diagnosed at an intermediate or advanced stage and are not surgical candidates, transcatheter arterial chemoembolization (TACE) is the primary treatment option. Previous studies found that after two consecutive TACE sessions, 22.5% of patients had no objective response, attributed to TACE failing to produce complete necrosis of HCC (2, 3). Combining TACE with local ablation techniques such as microwave ablation, radiofrequency ablation, cryoablation, and high-intensity focused ultrasound (HIFU) has been shown to improve overall survival rates when compared to TACE alone (4–7).

For HCC, HIFU has proven a non-invasive therapy option (8). HIFU was described as a new ablative strategy for small liver cancer in the clinical practice guidelines of the European Association for the Study of the Liver (EASL) (9). HIFU is also regarded as a key therapeutic approach for ablation in the Medical Administration of the National Health and Health Commission of the People’s Republic of China guidelines for primary liver cancer (2019 edition) (10). TACE in combination with HIFU, on the other hand, has not been recommended by any guidelines for intermediate or advanced HCC. This is most likely due to the fact that HIFU is still in its infancy and its efficacy has yet to be validated (11).

Several studies have investigated the impact of combining TACE and HIFU in patients with intermediate and advanced HCC when compared to TACE alone (7, 11–20). However, these studies did not show consistent conclusion that TACE in combination with HIFU has a better overall survival or tumor response than TACE alone. Therefore, a meta-analysis is necessary to comprehensively demonstrate the efficacy of TACE in conjunction with HIFU in HCC.

In this study, we intended to conduct a meta-analysis by searching multiple online databases thoroughly. In addition, we performed subgroup analyses based on variables such as sample size, age, and tumor size to explore whether the conclusion is valid. This meta-analysis utilizes the primary outcomes of overall survival and tumor response to evaluate if TACE in conjunction with HIFU is more effective than TACE alone in the management of intermediate and advanced HCC. This study was conducted in accordance with the guidelines for the “Preferred Reporting Items for Systematic Reviews and Meta-Analyses (PRISMA)” (21).

## Materials and Methods

### Search Strategy

The protocol of this meta-analysis was registered on the international prospective register of systematic reviews database (PROSPERO: CRD42020203484). PubMed, Embase, Cochrane Library, Web of Science, Wanfang Data, CQVIP, China National Knowledge Infrastructure (CNKI), and Chinese Biomedical (CBM) databases were searched for randomized controlled trials (RCTs) that compared the effects of TACE in combination with HIFU and TACE alone in treating HCC that were published before October 6, 2021. Medical subject headings (MeSH) and free words were combined for literature retrieval. We mainly used the following search terms: “HIFU”, “high-intensity focused ultrasound”, “focused ultrasound”, “FUAS”, “focused ultrasound ablation surgery”, “TACE”, “Transarterial chemoembolization”, “HCC”, and “hepatocellular carcinoma”. No language was limited during the literature search. Institutional Review Board (IRB) approval and written consent were not required for conducting this meta-analysis.

### Inclusion and Exclusion Criteria

Inclusion criteria: 1) studies where the patients were diagnosed with primary intermediate or advanced HCC. The original study should demonstrate that patients with intermediate or advanced liver cancer were included. The diagnostic criterion, which could be TNM or BCLC grade, was not restricted. 2) Studies where patients in the TACE combined with HIFU group (group A) received HIFU after TACE treatment, whereas patients in the TACE alone group (group B) received only TACE. 3) Studies where any of the primary or secondary outcomes was reported. The primary outcomes were the 6-month overall survival (OS) rate, 12-month OS rate, 24-month OS rate, and tumor response. OS was defined as the period from the date of certain treatment to the date of death from any cause. Tumor response was evaluated according to WHO criterion, RECIST criterion, RECIST 1.1 criterion, modified RECIST criterion, or other criteria. Tumor response was usually assessed one month after treatment. Each criterion included the classification of complete response (CR), partial response (PR), stable disease (SD), and progressive disease (PD). Tumor response was reflected by overall response rate, which was calculated using the formula “CR+PR”. Post-treatment complication was the secondary outcome. 4) Only RCTs were considered for this study. Exclusion criteria: 1) The full text was not available; 2) the study belonged to animal experiment; 3) the study was not related to our subject; or 4) the study used other therapies that were combined with group A or group B.

### Study Selection, Data Extraction, and Assessment of Methodological Quality

Two reviewers (YBW and RM) examined the full texts independently and extracted the data. Any disagreements among reviewers were resolved by consulting with another senior coauthor. We collected the following data: first author, publication year, region, study design, intervention technique, sample size, age, gender, Child-pugh grade, clinical stage, tumor size, percentage of single tumor, and outcomes. The Cochrane handbook was utilized to assess the methodological quality of the included RCTs (22).

### Statistical Analysis

When the survival rate for specific months in a study was not available but the survival curve was provided, the survival rate was calculated using Engauge Digitizer software (version 10.8). The pooled value was calculated using the Mantel-Haenszel method as well as the study-specific odds ratio (OR) and 95% confidence interval (CI) for the categorical variables. When significant statistical heterogeneity was identified, the outcomes were combined using random effects model. Otherwise, the fixed effects model would be employed. Stata software (version 16.0) was used for data synthesis. Heterogeneity between different studies was evaluated by the I^2^ statistic and the chi-squared test. When P < 0.05, significant heterogeneity was identified. Furthermore, I^2^ value ≤ 50%, 50% < I^2^ value ≤ 75%, and I^2^ value > 75% were considered to be low, moderate, and high heterogeneity, respectively. When high heterogeneity was detected, the potential origins would be explored. Sensitivity analysis was performed using the “leave one out” method. Publication bias was evaluated using Begg’s test and Egger’s test and was shown by funnel plot. P < 0.05 was considered statistically significant.

## Results

### Characteristics of the Included Studies

**Figure 1:**
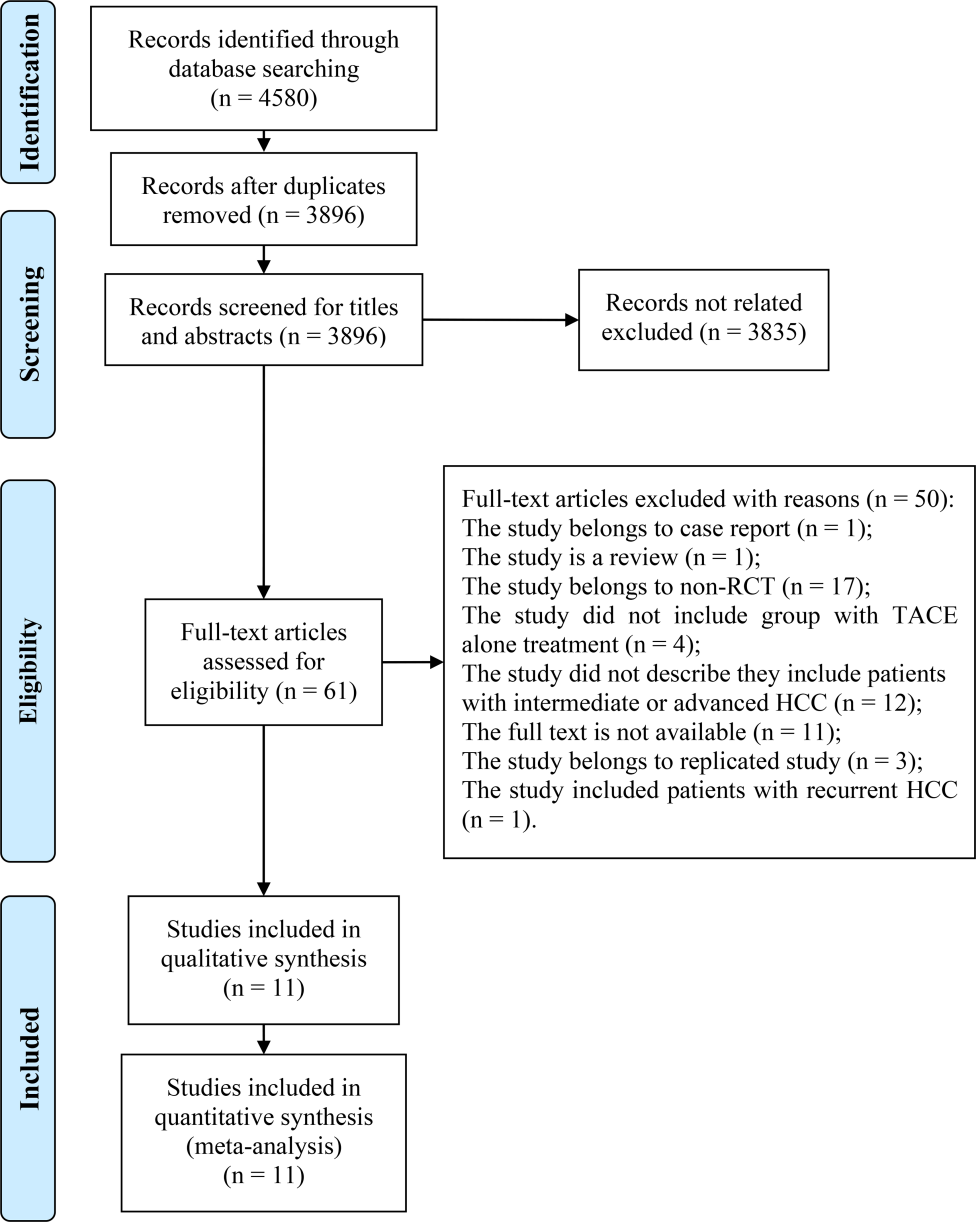
Flow diagram of literature selection. A flow diagram of the literature selection process is shown. We found 4580 citations after searching eight online databases. The titles and abstracts were then reviewed for relevance. We identified 61 citations and reviewed them using their full-texts. Finally, for qualitative and quantitative synthesis, we included eleven RCTs. RCT, randomized controlled trial; TACE, transcatheter arterial chemoembolization; HCC, hepatocellular carcinoma.

We obtained 4580 citations after performing a literature search. We started by removing duplicate studies, retaining 3896 citations. Next, we further excluded 3835 citations after we screened the titles and abstracts for relevance, yielding 61 citations that were reviewed for further consideration. Finally, for quantitative synthesis, 11 RCTs (7, 11–20) that fit the inclusion criteria of this meta-analysis were identified. Literature selection is summarized in **Figure 1**.

The included studies were published between 2005 and 2019. When combined, our study included 399 patients in group A and 404 patients in group B. In group A, HIFU ablation was conducted after TACE treatment. Six of the 11 RCTs (11, 13–15, 18, 19) identified the time interval between HIFU and TACE, approximately 2-4 weeks. One study set the time interval as one week (7). Four studies (12, 16, 17, 20) did not report the time interval. All study provided the information about the age and sex. Eight of the elven studies reported that they included patients with mean age >52. Among the 11 RCTs, nine RCTs included intermediate and advanced HCC, and the remaining two studies included advanced HCC. Seven RCTs said they used TNM stage, and four studies did not report the criteria they used. Furthermore, eight RCTs reported the Child-Pugh score, while three studies did not. Seven of the eight studies showed that they included patients with Child-pugh A or B. Only one study included patients with Child-pugh C in both groups. The detailed characteristics of the included studies are shown in **Table 1**.

**Table 1:** Characteristics of the included studies.

First author (Year)	Group	No. of patients	Age, y, mean (SD^a^)	Sex (Male/Female)	Child-pugh grade (A/B/C)	Clinical stage for all patients in each study	Tumor size, cm, mean (SD^a^)	Single tumor, %	6-,12-,24-months OS rate	CR/PR/SD^b^/PD
Wu F (2005)(19)	A	24	47 ± 12.6	15/9	24/0/0	Advanced HCC (TNM stage IVa)	10.03(No SD^a^)	25.00	80.15^c^ (80.4-85.4)^d^/42.9/NA	All patients: NA
	B	26	44.5 ± 8.4	21/5	24/2/0		11.26 (No SD^a^)	34.62	13.2/0/NA	
Chen WZ (2005) (18)	A	61	52.5 ± 13.1	49/12	59/2/0	Intermediate and advanced HCC (TNM stage III and IV)	9.8 ± 2.9	All patients: NA	82.41/65.14/31.37	All patients: NA
	B	66	53.4 ± 13.6	55/11	65/1/0		9.4 ± 2.8		44.42/12.48/6.2	
Cao W (2009) (17)	A	30	All patients: 40.9 (No SD^a^)	All patients: 43/17	All patients: 18/42/0	Intermediate and advanced HCC (TNM stage II, III, and IV)	All patients: 3.9 (No SD^a^)	All patients: NA	All patients: NA	3/18/8/1
	B	30								1/12/12/5
Li P (2013) (15)	A	25	59.40 ± 11.79	22/3	17/8/0	Intermediate and advanced HCC (TNM stage III and IV)	All patients: NA	All patients: NA	72/59.1/NA	1/20/2/2
	B	22	58.27 ± 12.15	18/4	11/11/0				48/31.8/NA	0/14/2/6
Du JK (2013) (16)	A	34	56(No SD^a^)	21/13	All patients: A or B	Intermediate and advanced HCC (no criteria reported)	All patients: NA	All patients: NA	100/94.12/52.94	3/21/10/1
	B	34	53(No SD^a^)	19/15					91.12/76.47/35.29	0/11/18/5
Dong WH (2015) (14)	A	34	60.5 ± 7.6	30/4	21/13/0	Intermediate and advanced HCC (TNM stage III and IV)	All patients: NA	All patients: NA	79.4/76.5/NA	2/27/2/3
	B	31	61.3 ± 9.2	28/3	16/15/0				54.8/51.6/NA	1/18/5/7
Fu SY (2015) (13)	A	36	All patients: 57.32(median)	All patients: 40/36	All patients: 56/20/0	Intermediate and advanced HCC (TNM stage III and IV)	All patients: 2.5-11.0(range)	All patients: NA	94.4/66.7/36.1	All patients: NA
	B	40							82.5/47.5/15	
Wang RJ (2018) (12)	A	30	53.5 ± 13.6	19/11	All patients: NA	Intermediate and advanced HCC (no criteria reported)	All patients: NA	All patients: NA	All patients: NA	4/18/7/1
	B	30	53.4 ± 12.5	20/10						0/10/17/3
Luo Y (2019) (11)	A	45	All patients: 58.34 ± 2.95	All patients: 52/38	All patients: NA	Intermediate and advanced HCC (no criteria reported)	All patients: 11.16 ± 3.28	All patients: NA	All patients: NA	15/23/5/2
	B	45								6/22/11/6
Zhang Q (2019) (7)	A	50	56 ± 11	25/25	9/20/21	Intermediate and advanced HCC (TNM stage II, III, IV)	All patients: NA	All patients: NA	96.70^c^/92.57^c^/84.17^c^	20/25/5/0
	B	50	55 ± 10	26/24	10/19/21				89.70^c^/85.98^c^/70.91^c^	15/15/10/0
Liang W (2018) (20)	A	30	53.5 ± 13.6	19/11	All patients: NA	Advanced HCC (no criteria reported)	All patients: NA	All patients: NA	All patients: NA	4/18/7/1
	B	30	53.4 ± 12.5	20/10						0/10/17/3

^a^The SD means standard deviation; ^b^The SD means one of the tumor response, which is stable disease; ^c^The OS rate was calculated by our study; ^d^The range was reported by the original study; Group A, TACE in combination with HIFU; Group B, TACE alone; TACE, transcatheter arterial chemoembolization; HIFU, high-intensity focused ultrasound; CR, complete response; PR, partial response; PD, progressive disease; NA, not available; OS, overall survival; HCC, hepatocellular carcinoma.

Methodological quality of the RCTs is shown in **Supplementary Figure 1**. As indicated, five RCTs (12, 13, 16, 19, 20) reported random sequence generation methods. All trials used randomization, but no strategies for allocation concealment were reported. As a result, the possibility of selection bias in most studies is regarded to be uncertain. One study by Wu F et al. (19) reported that the operator who performed TACE was blinded, but other operators as well as participants were not. Furthermore, as other studies did not report that they blinded participants and personnel, the risk of performance bias for all studies is high. Only the study by Wu F et al. (19) blinded the outcome assessment, so the risk of detection bias for all studies is high. As four of the studies did not specify whether or not follow-up was completed, the risk of attrition bias is undetermined. No study was found to have selective reporting, so the risk of reporting bias is low. Additionally, no other bias was found.

### Meta-Analysis of Overall Survival

**Figure 2:**
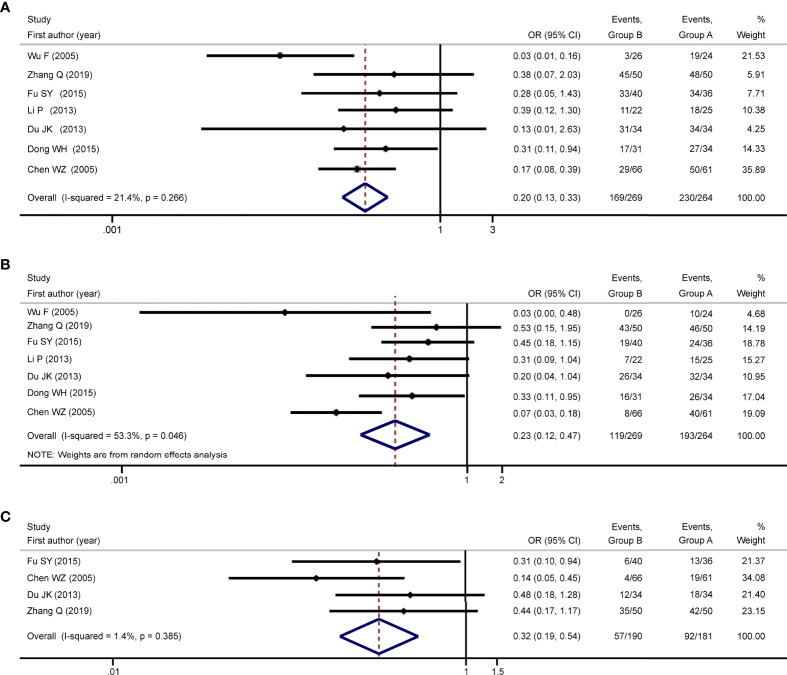
Meta-analysis of overall survival. Meta-analysis of overall survival was conducted with the outcomes of 6-month OS rate, 12-month OS rate, and 24-month OS rate, respectively. Results of the meta-analyses showed that group A was associated with increased 6-month OS rate [OR: 0.20; 95% CI: 0.13-0.33; **(A)**], 12-month OS rate [OR: 0.23; 95% CI: 0.12-0.47; **(B)**], and 24-month OS rate [OR: 0.32; 95% CI: 0.19-0.54; **(C)**] compared to group B, and no high statistical heterogeneities were detected. Group A: TACE in combination with HIFU; Group B: TACE alone; TACE, transcatheter arterial chemoembolization; HIFU, high-intensity focused ultrasound; OS, overall survival; OR, odds ratio; CI, confidence interval.

The 6-month OS rate in group A (87.12%) was significantly higher than that in group B (62.83%) [OR = 0.20; 95% CI = 0.13 to 0.33; P < 0.001; **Figure 2A**], with low heterogeneity (P = 0.27; I^2^ = 21.4%), according to the meta-analysis of seven studies (7, 13–16, 18, 19). This difference was supported by subgroup analyses based on sample size, age, and tumor size (**Supplementary Table 1**). Furthermore, a meta-analysis of seven studies (7, 13–16, 18, 19) revealed that the 12-month OS rate in group A (73.11%) was significantly higher than that in group B (44.24%) [OR = 0.23; 95% CI = 0.12 to 0.47; P < 0.001; **Figure 2B**], with moderate heterogeneity (P = 0.046; I^2^ = 53.3%). This difference was again supported by subgroup analyses based on sample size, age, and tumor size (**Supplementary Table 1**). Additionally, meta-analysis of four studies (7, 13, 16, 18) showed that the 24-month OS rate in the group A (50.83%) was significantly higher than that in the group B (30.0%) [OR = 0.32; 95% CI = 0.19 to 0.54; P < 0.001; **Figure 2C**], with low heterogeneity (P = 0.39; I^2^ = 1.4%). The result of subgroup analyses based on different sample size and age supported this difference (**Supplementary Table 1**).

### Meta-Analysis of Tumor Response

Among the eleven studies included, one study (18) did not report the outcome of tumor response, four studies (12, 16, 17, 20) reported tumor response based on WHO criterion, three studies (11, 14, 15) reported tumor response using the RECIST criterion, one study (7) reported tumor response using the modified RECIST criterion, and two studies (13, 19) reported tumor response using other criteria. Considering that different criteria defined the tumor response differently, we performed a meta-analysis based on each reported criterion.

**Figure 3:**
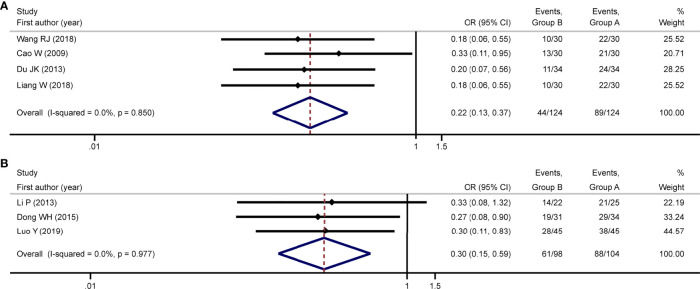
Meta-analysis of tumor response. Meta-analysis of tumor response was conducted using studies with WHO criterion and RECIST criterion, respectively. Using studies reporting tumor response with WHO criterion, the meta-analysis found group A was associated with improved overall response rate compared to group B [OR: 0.22; 95% CI: 0.13-0.37; **(A)**]. Using studies reporting tumor response with RECIST criterion, the meta-analysis found group A was associated with improved overall response rate compared to group B [OR: 0.30; 95% CI: 0.15-0.59; **(B)**]. No heterogeneity was detected in either meta-analysis. Group A: TACE in combination with HIFU; Group B: TACE alone; TACE, transcatheter arterial chemoembolization; HIFU, high-intensity focused ultrasound; OR, odds ratio; CI, confidence interval.

Meta-analysis of four studies (12, 16, 17, 20) using WHO criterion showed that the overall response rate in the group A (71.77%) was significantly higher than that in the group B (35.48%) (OR = 0.22; 95% CI = 0.13 to 0.37; P < 0.001; **Figure 3A**), with no heterogeneity (P = 0.85; I^2^ = 0). The result of subgroup analyses based on sample size < 70 and age < 57 supported this difference (**Supplementary Table 2**). Meta-analysis of three trials (11, 14, 15) using RECIST criterion showed that the overall response rate in the group A (84.62%) was significantly higher than that in the group B (62.24%) (OR = 0.30; 95% CI = 0.15 to 0.59; P < 0.001; **Figure 3B**), with no heterogeneity (P = 0.98; I^2^ = 0). The result of subgroup analyses based on sample size < 70 and age ≥ 57 supported this difference (**Supplementary Table 2**).

### Posttreatment Complications

The posttreatment complications from each study were extracted and summarized in **Supplementary Table 3**. As shown, one study (11) reported two serious complications: digestive tract hemorrhage and renal failure. The group A was associated with a lower percentage of digestive tract hemorrhage compared to the group B (P = 0.049). However, renal failure showed no difference between the two groups. No other studies reported serious complications. In the group A, some mild complications, such as fever, skin burn, mild local pain, and subcutaneous edema, were reported in these studies. These mild complications usually rapidly resolved within 3-7 days after HIFU treatment without special treatment. No additional meta-analysis was performed due to the limited number of serious complications reported.

### Sensitivity Analyses

Sensitivity analyses were conducted on 6-month OS rate, 12-month OS rate, 24-month OS rate, and overall response rate (with WHO criterion and RECIST criterion). Utilizing the “leave one out” method, we found that the difference in any meta-analysis between group A and group B was still statistically significant and had not been changed.

### Publication Bias

**Figure 4:**
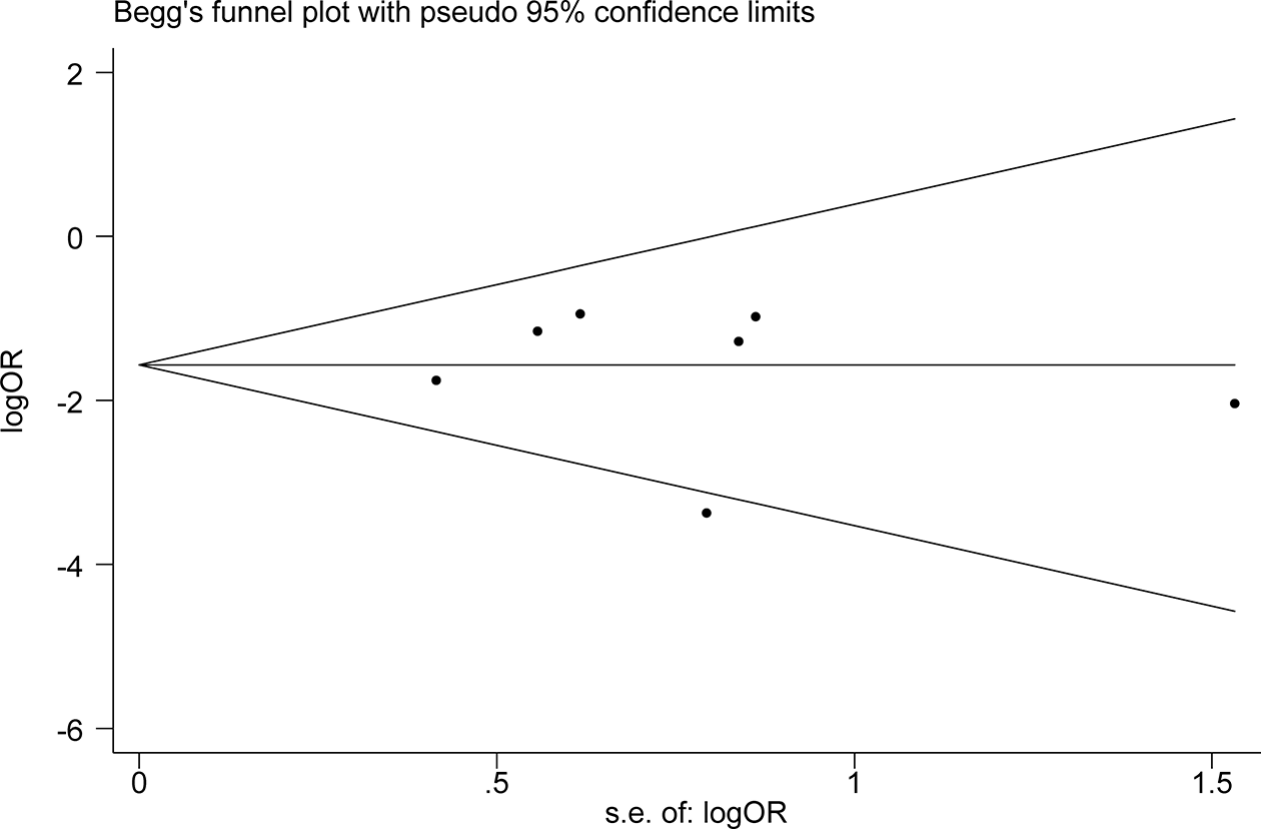
Evaluation of publication bias. Begg’s funnel plot was used to detect publication bias in 6-month OS rate. Each dot in the funnel plot represents a study. Those points are symmetrical on both sides of the reference line, indicating no publishing bias. OS, overall survival; OR, odds ratio.

To evaluate publication bias, the outcome of the 6-month OS rate was used. Begg’s test (P=1.00), Egger’s test (P=0.82), and the Begg’s funnel plot (**Figure 4**) all indicate that there was no publication bias. Each dot in the funnel plot represents a study. As shown in the figure, the points are symmetrical on both sides of the reference line.

## Discussion

HIFU was first proposed for treatment in 1932, when Freundlich H, Collner K, and Rogowski F found the medium’s propensity to heat tissue (23). The JC HIFU tumor treatment system was first developed and utilized in clinic by the Ultrasound Institute of Chongqing Medical University in 1997. HIFU is a non-invasive technique of local thermal ablation. Its basic premise is to focus low-energy ultrasound *in vitro* on the target tissue *in vivo*, resulting in coagulative necrosis *via* ultrasound’s biological effects such as thermal effect, cavitation effect, and mechanical impact (24). At present, HIFU technology is mainly used in benign and malignant solid tumors and benign diseases of uterus, prostate and other organs. As HIFU can ablate the local tumor while being monitored through ultrasound or MRI, it is considered both safe and accurate. When compared to traditional surgical resection, HIFU technology is minimally invasive, therefore it can be utilized as an alternate treatment when traditional surgery is not feasible.

Our meta-analysis found that the 6-month, 12-month, and 24-month OS in group A were significantly better compared to group B. The meta-analysis also indicated that group A was associated with increased overall response rate compared to group B. Therefore, our meta-analysis found that HIFU combined with TACE had better short-term and long-term efficacy than TACE alone. Furthermore, the heterogeneity of the meta-analysis for each outcome was not high. The result of subgroup analyses based on different sample size, age, and tumor size was consistent with the result of the meta-analyses including all studies. In addition, sensitivity analyses found the result of the meta-analyses was not influenced by any single study. Additionally, our study identified no evidence of publication bias, implying that the literature search was comprehensive. These additional analyses, taken collectively, imply that the conclusion of our meta-analysis is reliable.

In our meta-analysis, we summarized the incidence of complications in both groups. Common complications induced by HIFU included fever, skin burn, mild local pain, and subcutaneous edema, which rapidly resolved 3-7 days after HIFU treatment without special treatment (25). It is worth noting that HIFU may also cause severe complications, such as bleeding and renal failure. However, the incidence rate of these severe complications is very low (26). Of note, among the included studies, two serious complications (renal failure and digestive tract hemorrhage) were reported in one study (11). For the incidence rate of renal failure, group A (n=1) and group B (n=0) showed no difference. However, for digestive tract hemorrhage, group B (n=6) exhibited a higher incidence rate compared to group A (n=1). The reason behind this is unexplained in the original study, and it may need to be investigated further in the future. In any case, our data suggested that TACE in conjunction with HIFU is safe for patients with intermediate and advanced HCC.

TACE is a major treatment for intermediate and advanced liver cancer. TACE has the ability to obstruct the arterial blood supply of liver cancer cells. Liver cancer, however, has a dual blood supply from the hepatic artery and the portal vein. In addition, the tumor may develop neovascularization and collateral circulation. These factors lead to incomplete tumor necrosis and affect the efficacy of TACE. In order to kill tumor cells as much as possible, TACE treatment often needs to be carried out many times. Repeated TACE could lead to chemotherapeutic cytotoxicity and aggravate the fibrosis progression, thus leads to the deterioration of liver function (27). When combined with other treatment methods, a synergistic anticancer effect can be achieved, and the survival time of patients can be prolonged as much as possible. A single treatment is frequently insufficient to achieve a satisfactory curative effect. More and more patients are opting for a multidisciplinary combination treatment (28). TACE treatment is an integral part of this multidisciplinary approach. At present, TACE therapy has been reported to be combined with HIFU, radiofrequency ablation, radiotherapy, targeted therapy and immunotherapy to improve the curative effect.

In this meta-analysis, we found that the combination of TACE and HIFU was better than TACE alone in the treatment of intermediate and advanced liver cancer. TACE’s therapeutic impact can be enhanced by HIFU, which may be due to the following processes. First, HIFU can induce tumor coagulative necrosis, which can enhance the death of localized tumor cells following TACE treatment and consolidate the therapeutic efficacy of TACE (29). Second, after TACE treatment, liver cancer cells near the portal vein may remain, and HIFU helps to eliminate these residual tumor cells. Furthermore, HIFU aids in the exposure of tumor antigens and the induction of an anti-tumor immune response, which may improve the efficacy of liver cancer treatment (30). Considering the role of HIFU after TACE treatment, the findings of our study showed that in clinical practice, if possible, combination with HIFU should be promoted for patients with intermediate and advanced HCC, rather than consecutive TACE.

Our research has some limitations. First, despite the fact that our study solely included RCTs, there were certain bias risks. For instance, because blinding of participants and personnel, as well as blinding of outcome assessment, are difficult to implement, performance bias and detection bias are difficult to avoid. Second, despite our best efforts to incorporate studies from various countries, all of the included studies identified were from China. This could be due to a variety of factors, including: 1) China had a high HCC disease burden, with many patients diagnosed with intermediate or advanced HCC (31); and 2) China developed and applied the JC HIFU system in clinic early, which has been subsequently recommended for the treatment of HCC. Whether TACE in combination with HIFU benefits patients from other countries as well still needs to be validated by further studies. Third, some details about TACE or HIFU therapy were not explored in this meta-analysis. The primary reason was due to limited information being reported in the original studies. More information, such as the frequencies of TACE or HIFU, the time spent on treatment, and the time interval between TACE and HIFU, are hoped to be reported and studied in future research. Furthermore, whether a single or multiple lesions were treated is critical for tumor treatment. In the study conducted by Wu F, et al, the entire tumors in combination group were treated with HIFU. According to another study conducted by Cao W, et al, a number of the patients did not achieve complete tumor ablation. The reasons mentioned were the tumor overlaps with the ribs, is adjacent to or invades the hepatic duct or gallbladder, and so cannot be completely ablated. For other studies, whether single lesions were treated or multiple was not reported in detail. Nevertheless, given that additional tumor ablation can lessen a patient’s tumor load and prolong the patient’s life, it could be argued that tumors in certain patients should be treated as much as possible. In any case, it is expected that future study should focus on how many lesions were treated.

Fourth, our study did not use hazard ratio (HR) as the effect size, but used OR instead. The main reason is that HR in most studies was not provided. So, to better evaluate the survival benefit from HIFU, further original studies would better consider HR as the effect size. Furthermore, the number of studies included in the meta-analysis is limited. We intended to incorporate as many studies as possible by searching all literature libraries recognized by academia. After completing our manuscript, we revisited our literature search by rescanning these databases. However, only the initial eleven studies were subsequently identified. Although the number of studies is limited, the results are reliable. The findings are useful for guiding clinical treatment. This meta-analysis could be updated when new studies are released in the future.

## Conclusion

TACE in combination with HIFU is associated with increased OS and tumor response compared to TACE alone in patients with intermediate and advanced HCC. The use of HIFU after TACE treatment in intermediate and advanced HCC is supported by current evidence.

## Data Availability Statement

The original contributions presented in the study are included in the article/**Supplementary Material**. Further inquiries can be directed to the corresponding authors.

## Ethics Statement

Ethical review and approval was not required for the study on human participants in accordance with the local legislation and institutional requirements. Written informed consent for participation was not required for this study in accordance with the national legislation and the institutional requirements.

## Author Contributions 

Conception and design: Y-BW, R M, Z-BW, Q-LS, J-PG, and JB. Collection and assembly of data: Y-BW and RM. Data analysis and interpretation: All authors. Manuscript writing: All authors. J-PG and JB contributed equally to this work. All authors contributed to the article and approved the submitted version.

## Funding

The study was funded by Chongqing medical scientific research project (Joint project of Chongqing Health Commission and Science and Technology Bureau: No. 2021MSXM139).

## Conflict of Interest

The authors declare that the research was conducted in the absence of any commercial or financial relationships that could be construed as a potential conflict of interest.

## Publisher’s Note

All claims expressed in this article are solely those of the authors and do not necessarily represent those of their affiliated organizations, or those of the publisher, the editors and the reviewers. Any product that may be evaluated in this article, or claim that may be made by its manufacturer, is not guaranteed or endorsed by the publisher.

## Abbreviations

HCC, Hepatocellular carcinoma; TACE, Transcatheter arterial chemoembolization; HIFU, High-intensity focused ultrasound; EASL, European association for the study of the liver; PRISMA, Preferred reporting items for systematic reviews and meta-analyses; CNKI, China national knowledge infrastructure; CBM, Chinese biomedical; RCTs, Randomized controlled trials; MeSH, Medical subject headings; IRB, Institutional review board; OS, Overall survival; CR, Complete response; PR, Partial response; SD, Stable disease; PD, Progressive disease; OR, Odds ratio; CI, Confidence interval; HR, Hazard ratio.
