# Supplementary material for: Transcatheter Arterial Chemoembolization in Combination With High-Intensity Focused Ultrasound for Intermediate and Advanced Hepatocellular Carcinoma: A Meta-Analysis
Source: Front Oncol. 2022 Mar 28;12:797349. doi: 10.3389/fonc.2022.797349 (PMC8999843; doi:10.3389/fonc.2022.797349)
Supplement: Supplementary file 1 [file DataSheet_1.docx]

Supplementary Material


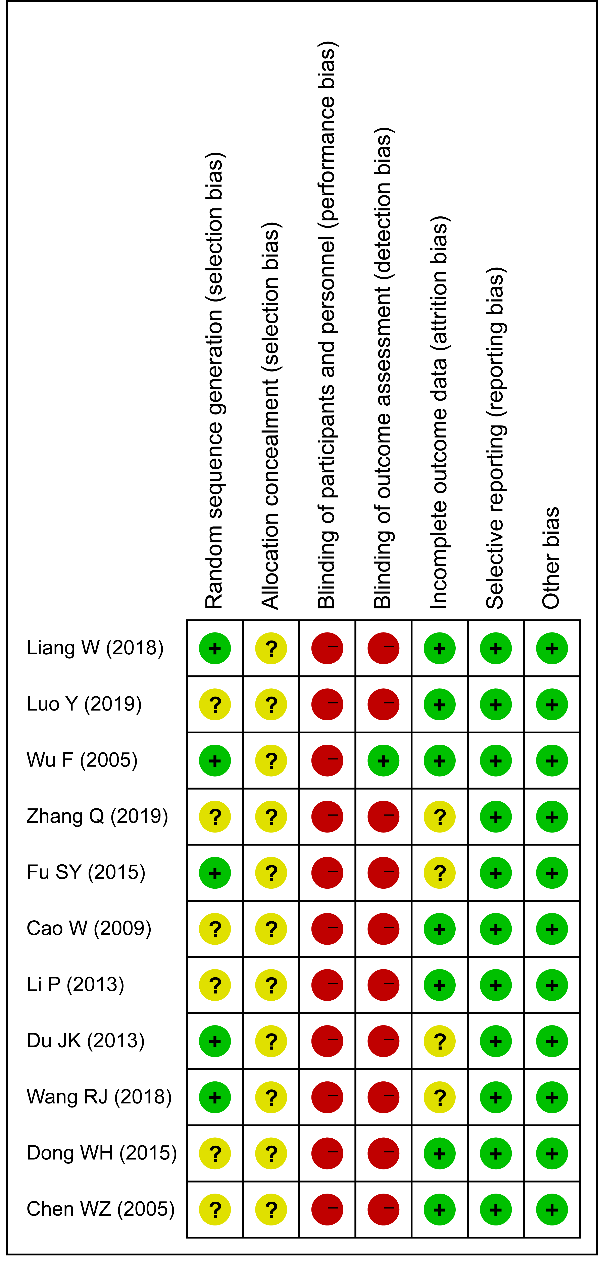


**Supplementary Figure 1. Methodological quality of the RCTs**

**Note:** The methodological quality of the RCTs was evaluated using the Cochrane handbook. As shown, the risk of selection bias for most studies is thought to be unclear. Blinding of participants and personnel was not well-conducted in most studies, so the risk of performance bias is high. Only one study blinded the outcome assessment, so the risk of detection bias for most studies is also high. Four studies did not describe whether follow-up was completed, so the risk of attrition bias is unclear. Additionally, the risk of reporting bias and other types of bias was not found.
