# Supplementary material for: Transcatheter Arterial Chemoembolization in Combination With High-Intensity Focused Ultrasound for Intermediate and Advanced Hepatocellular Carcinoma: A Meta-Analysis
Source: Front Oncol. 2022 Mar 28;12:797349. doi: 10.3389/fonc.2022.797349 (PMC8999843; doi:10.3389/fonc.2022.797349)
Supplement: Supplementary file 2 [file Table_1.docx]

Supplementary Material

**Supplementary Table 1. Subgroup analyses of overall survival**

| Outcome | Subgroup | Classification | Study number | OR (95%CI) | P for effect | I^2^ (%) | P for heterogeneity |
| --- | --- | --- | --- | --- | --- | --- | --- |
| 6-month OS | Total |  | 7 | 0.20 (0.13, 0.33) | <0.001 | 21.4 | 0.27 |
|  | Sample size | <70 | 4 | 0.20 (0.10, 0.38) | <0.001 | 56.2 | 0.08 |
|  |  | ≥70 | 3 | 0.21 (0.11, 0.42) | <0.001 | 0 | 0.67 |
|  | Age (mean) | <57 | 4 | 0.14 (0.08, 0.27) | <0.001 | 36.1 | 0.20 |
|  |  | ≥57 | 2 | 0.35 (0.15, 0.78) | 0.010 | 0 | 0.80 |
|  | Tumor size (mean) | ≥9.4 | 2 | 0.12 (0.06, 0.24) | <0.001 | 69.2 | 0.07 |
| 12-month OS | Total |  | 7 | 0.23 (0.12, 0.47) | <0.001 | 53.3 | 0.046 |
|  | Sample size | <70 | 4 | 0.22 (0.11, 0.43) | <0.001 | 0 | 0.40 |
|  |  | ≥70 | 3 | 0.25 (0.07, 0.94) | 0.040 | 79.7 | 0.01 |
|  | Age (mean) | <57 | 4 | 0.13 (0.07, 0.24) | <0.001 | 60.6 | 0.06 |
|  |  | ≥57 | 2 | 0.32 (0.15, 0.71) | 0.005 | 0 | 0.95 |
|  | Tumor size (mean) | ≥9.4 | 2 | 0.06 (0.03, 0.15) | <0.001 | 0 | 0.50 |
| 24-month OS | Total |  | 4 | 0.32 (0.19, 0.54) | <0.001 | 1.4 | 0.39 |
|  | Sample size | ≥70 | 3 | 0.28 (0.15, 0.51) | <0.001 | 11.0 | 0.33 |
|  | Age (mean) | <57 | 3 | 0.33 (0.18, 0.58) | <0.001 | 34.0 | 0.22 |

**Note**: The subgroup with only one study was not analyzed and shown. OR: odds ratio; CI: confidence interval; OS: overall survival.
