# Supplementary material for: Transcatheter Arterial Chemoembolization in Combination With High-Intensity Focused Ultrasound for Intermediate and Advanced Hepatocellular Carcinoma: A Meta-Analysis
Source: Front Oncol. 2022 Mar 28;12:797349. doi: 10.3389/fonc.2022.797349 (PMC8999843; doi:10.3389/fonc.2022.797349)
Supplement: Supplementary file 3 [file Table_2.docx]

Supplementary Material

**Supplementary Table 2. Subgroup analyses of tumor response**

| Criterion | Outcome | Classification | Subgroup | Study number | OR (95%CI) | P for effect | I^2^ (%) | P for heterogeneity |
| --- | --- | --- | --- | --- | --- | --- | --- | --- |
| WHO | Overall response rate |  | Total | 4 | 0.22 (0.13, 0.37) | <0.001 | 0 | 0.85 |
|  |  | Sample size | <70 | 4 | 0.22 (0.13, 0.37) | <0.001 | 0 | 0.85 |
|  |  | Age (mean) | <57 | 4 | 0.22 (0.13, 0.37) | <0.001 | 0 | 0.85 |
| RECIST | Overall response rate |  | Total | 3 | 0.30 (0.15, 0.59) | <0.001 | 0 | 0.98 |
|  |  | Sample size | <70 | 2 | 0.30 (0.12, 0.73) | 0.008 | 0 | 0.83 |
|  |  | Age (mean) | ≥57 | 3 | 0.30 (0.15, 0.59) | <0.001 | 0 | 0.98 |

**Note**: The subgroup with only one study was not analyzed and shown. OR: odds ratio; CI: confidence interval.
