# Supplementary material for: Transcatheter Arterial Chemoembolization in Combination With High-Intensity Focused Ultrasound for Intermediate and Advanced Hepatocellular Carcinoma: A Meta-Analysis
Source: Front Oncol. 2022 Mar 28;12:797349. doi: 10.3389/fonc.2022.797349 (PMC8999843; doi:10.3389/fonc.2022.797349)
Supplement: Supplementary file 4 [file Table_3.docx]

Supplementary Material

**Supplementary Table 3. Summary of the posttreatment complications**

| First author | Group A | Group B |
| --- | --- | --- |
| Luo Y [11] | Digestive tract hemorrhage (1), Renal failure (1), Fever (3), Nausea and vomiting (4) | Digestive tract hemorrhage (6), Renal failure (0), Fever (4), Nausea and vomiting (5) |
| Wu F [19] | After HIFU:  Fever (1), Skin burn (2), Mild local pain (4)  For all patients after TACE:  Transient impairment of hepatic function (all), Transient fever (45), Transient pain (30) | |
| Zhang Q [7] | Jaundice (1), Ascites (1), Gastrointestinal reactions (2), Liver discomfort (1) | Jaundice (5), Ascites (8), Gastrointestinal reactions (8), Liver discomfort (8) |
| Cao W [17] | NA | NA |
| Chen WZ [18] | NA | NA |
| Dong WH [14] | Mild to moderate subcutaneous edema (3), Mild to moderate fever (12)，Skin burn (1) | Pain of liver region (7)，Transaminase increased (6) |
| Du JK [16] | For both groups：  The symptoms of post-embolism syndrome such as fever, vomiting and pain (usually); Transient liver dysfunction (all) | |
| Fu SY [13] | For all patients:  Mild to moderate fever (13), Right upper abdominal pain (5), Hiccup, nausea and vomiting (5) | |
| Li P [15] | After HIFU:  Mild to moderate subcutaneous edema (all), Mild to moderate fever (10), Mild to moderate skin burn and pain in the operation area (1)  For all patients after TACE:  Post-embolism syndrome, such as different degrees of fever, nausea, liver discomfort, abdominal distension, high level of transaminase (all), Mild numbness of hands and feet (a few patients) | |
| Wang RJ [12] | Vomiting (1), Increased heart rate (1), Fever (1) | Vomiting (1), Fever (1) |
| Liang W [20] | Vomiting (1), Increased heart rate (1), Fever (1) | Fever (1), Vomiting (1) |

**Note**: Group A: TACE in combination with HIFU; Group B: TACE alone; TACE: transcatheter arterial chemoembolization; HIFU: high-intensity focused ultrasound; NA: not available. The number in the parenthesis “()” represents the cases of the corresponding complication.
